# Evaluation of triple negative breast cancer with heterogeneous immune infiltration

**DOI:** 10.3389/fimmu.2023.1149747

**Published:** 2023-05-04

**Authors:** Ángela Quintana, Enrique Javier Arenas, Cristina Bernadó, José Fernández Navarro, Jonatan González, Anna Esteve-Codina, Teresa Moliné, Merce Marti, Giuseppe Curigliano, Peter Schmid, Vicente Peg, Joaquín Arribas, Javier Cortés

**Affiliations:** ^1^ Breast Cancer Unit, Vall d’Hebrón Institute of Oncology, Barcelona, Spain; ^2^ Preclinical Research Program, Vall d’Hebron Institute of Oncology, Barcelona, Spain; ^3^ Biomedical Research Network Centre in Oncology (CIBERONC), Madrid, Spain; ^4^ Bioinformatics Department, Vall d’Hebrón Institute of Oncology, Barcelona, Spain; ^5^ CNAG-CRG, Centre for Genomic Regulation, Barcelona Institute of Science and Technology (BIST), Barcelona, Spain; ^6^ Universitat Pompeu Fabra (UPF), Barcelona, Spain; ^7^ Department of Pathology, Vall d’Hebron University Hospital, Barcelona, Spain; ^8^ Departament Biologia Cel·lular, Fisiologia i Immunologia, Universitat Autònoma de Barcelona, Barcelona, Spain; ^9^ European Institute of Oncology, IRCCS, Milan, Italy; ^10^ Department of Oncology and Hemato-Oncology, University of Milano, Milan, Italy; ^11^ Barts Cancer Institute, Queen Mary University London, London, United Kingdom; ^12^ Departamento de Medicina, Universitat Autonoma de Barcelona, Barcelona, Spain; ^13^ Departament de Medicina i Ciències de la Vida, Universitat Pompeu Fabra, Barcelona, Spain; ^14^ Catalan Institution for Research and Advanced Studies, (ICREA), Barcelona, Spain; ^15^ Cancer Research Program, Institut Hospital del Mar d’Investigacions Mèdiques (IMIM), Barcelona, Spain; ^16^ Universidad Europea de Madrid, Faculty of Biomedical and Health Sciences, Department of Medicine, Madrid, Spain; ^17^ International Breast Cancer Center, Pangaea Oncology, Quironsalud Group, Barcelona, Spain; ^18^ Medica Scientia Innovation Research (MedSIR), Barcelona, Spain

**Keywords:** tumor-infiltrating lymphocytes, intratumor heterogeneity, triple negative breast cancer, transcriptomics, immune cell abundance

## Abstract

**Introduction:**

Tumor infiltrating lymphocytes (TILs) are known to be a prognostic and predictive biomarker in breast cancer, particularly in triple negative breast cancer (TNBC) patients. International guidelines have been proposed to evaluate them in the clinical setting as a continuous variable, without a clear defined cut-off. However, there are scenarios where the immune infiltration is heterogeneous that some areas of the patient’s tumour have high numbers of TILs while other areas completely lack them. This spontaneous presentation of a heterogeneous immune infiltration could be a great opportunity to study why some tumours present TILs at diagnosis but others do not, while eliminating inter patient’s differences.

**Methods:**

In this study, we have identified five TNBC patients that showed great TIL heterogeneity, with areas of low (≤5%) and high (≥50%) numbers of TILs in their surgical specimens. To evaluate immune infiltration heterogeneity, we performed and analyzed bulk RNA-sequencing in three independent triplicates from the high and low TIL areas of each patient.

**Results:**

Gene expression was homogeneous within the triplicates in each area but was remarkable different between TILs regions. These differences were not only due to the presence of TILs as there were other non-inflammatory genes and pathways differentially expressed between the two areas.

**Discussion:**

This highlights the importance of intratumour heterogeneity driving the immune infiltration, and not patient’s characteristics like the HLA phenotype, germline DNA or immune repertoire.

## Introduction

Triple negative breast cancer (TNBC) is an aggressive breast tumor type in which the immune system plays an important role in its development and control. Despite many advancements in the recent years, chemotherapy is still the backbone of the treatment for these patients. Pembrolizumab, an immune checkpoint inhibitor (ICI) that blocks PD-1, combined with chemotherapy has proved to increase pathological complete response (pCR) and event free survival in localized TNBC, independently of the expression of PD-L1 (1, 2). In the metastatic setting, pembrolizumab in combination with different chemotherapies has also been approved, as it showed benefit in progression free survival and overall survival in PD-L1 positive TNBC patients (3). Several trials assessed the use of immunotherapy as monotherapy in metastatic patients without positive results (4, 5). Interestingly, ICIs have shown benefit in the window opportunity period but no therapy has been approved yet. One of these studies is the BELLINI trial, in which ipilimumab (aCTLA-4) and nivolumab (aPD-1) were evaluated before neoadjuvant chemotherapy or surgery. In this study, three patients had surgery after a few doses of these ICIs, one of them having pCR and another one near-pCR (6). Another study, the GeparNuevo, evaluated durvalumab (aPD-L1)/placebo in the window period followed by chemotherapy plus durvalumab/placebo as a neoadjuvant treatment. Only patients who received durvalumab in the window period had a statistically significant pCR response (61.0% vs 41.4%) (7).

Tumor-infiltrating lymphocytes (TILs) have been considered a potential biomarker that could predict response to neoadjuvant chemotherapy alone (8, 9) or combined with immunotherapy (6, 7, 10, 11), as well as prognosis in these patients (9, 12–17). Guidelines of how to score TILs have been published (18), where it is recommended to mainly report the score of stromal TILs (% of the stromal area). A recent pool analysis of TNBC, node negative patients that received chemotherapy and with ≥30% of TILs had excellent survival, suggesting that TILs can be used as a biomarker to identify a subset of patients with good prognosis that may need less or no chemotherapy (15). This cutoff (30%) was further assessed in another cohort of TNBC patients that did not received chemotherapy (only surgery with or without radiotherapy), and TILs proved to be a great biomarker to differentiate patients with good prognosis (19). It seems that in these patients, despite being able to elicit a strong immune response, the immune system could not completely eradicate the tumor but was able to control the tumor in its site of origin and created a long-lasting antitumor immunity memory in most of the patients, based on the low number of recurrences observed in the study.

In a small proportion of patients, immune infiltration can be highly heterogeneous, having some areas with little or no immune infiltration at all (≤5%) while other areas showing high immune infiltration (≥50%) (20). In these particular cases, the whole TILs score may not truly reflect the behavior of the tumor and how the immune system recognized it and attempted to eradicate it. However, having a patient that spontaneously develops a tumour where half of the area contains many lymphocytes while the other half does not might be an ideal scenario to study if the heterogenous biology of TNBC tumors might explain these two opposite cases of immune infiltration while eliminating the differences between patients. With only a few cases, we were still able to advance in the understanding of why some tumours are unable to trigger an immune response, since we are reducing the number of variables and co-founding factors that arise in an experiment when you compare different patients.

Most of the research has focused on studying the gene expression of patients with high TILs vs. low TILs, to understand why the lymphocytes in the first group were able to penetrate the tumour microenvironment while they did not in the other group. In this study, these two phenotypes are occurring within the same patient, meaning that it has the same HLA phenotype, germline DNA, and immune repertoire. This allows us to study the differences of immune infiltration with less patient-specific variables that can affect the conclusions. We hypothesize that this uneven heterogeneity in the immune infiltration within the same patient is caused by a differential transcriptomic landscape between the two tumor areas which could be explained by the tumour heterogeneity. Using whole RNA sequencing, we showed that there are transcriptomic differences between the low and high TIL regions, not only in pathways related to the immune system, but also in other oncogenic pathways.

## Methods

### Patients and samples

Samples and data from patients included in this study were obtained from the Pathological Anatomy Department of Vall d’Hebron Hospital, and were processed following standard operating procedures with the appropriate approval of the Ethical and Scientific Committees. Patients were identified during a previous study (21), in which we searched for pathology reports in the database to find patients diagnosed with TNBC until 2010, when neoadjuvant treatment was less common. 787 pathology reports were found, and the search was consequently followed by the review of the medical history to confirm patients that have localized tumors with no metastasis or lymph nodes affected at diagnosis (T1c-T2N0M0), and that have not received any treatment before surgery. 102 patients met the criteria, so for those with tumor block available at the hospital, TILs were assessed by two independent investigators following the international guidelines (18). We identified five patients that approximately half of the tumor was low TILs (≤5%) and the other half was high TILs (≥50%).

### RNA sequencing

Three punches per area of high and low TILs were performed using a 1.5mm punch sampling tool (BioPunch^®^). RNA was extracted from the formalin-fixed paraffin-embedded (FFPE) punches using Maxwell 16 LEV RNA FFPE kit and quantified by Qubit^®^ RNA BR Assay kit (Thermo Fisher Scientific). The RNA integrity and the quality metrics DV200 were estimated by using RNA 6000 Nano Bioanalyzer 2100 Assay (Agilent). The libraries were prepared using the KAPA RNA HyperPrep Kit with RiboErase (HMR) kit protocol (Roche Kapa), according to the manufacturer’s protocol with minor modifications. The libraries were sequenced on HiSeq 4000 (Illumina) with a read length of 2x76bp+8bp+8bp using HiSeq 4000 SBS kit (Illumina) and HiSeq 4000 PE Cluster kit (Illumina), following the manufacturer’s protocol for dual indexing. Image analysis, base calling and quality scoring of the run were processed using the manufacturer’s software Real Time Analysis (RTA 2.7.7).

### Bioinformatic analysis

Raw data was processed with the version 0.3.7 of an open-source pipeline available at https://github.com/jfnavarro/hla_pipeline using the reference genome GRC38h. In summary, each sample was quality-trimmed with trim-galore (22), aligned with STAR (23) and gene counts were derived using featureCounts (24). Only genes that were protein coding, with a total expression value greater than 10 and detected in at least 3 samples were kept resulting in a total of 19282 genes. Expression values were normalized using the variance-stabilizing transformation (VST) function of DESeq2 (25). Different analyses were performed to assess the quality of the samples as well as the possible batch effects (PCA, heatmap, pair-wise correlation and boxplots).

Differential expression analysis was performed using DESeq2 (25) with default settings. The contrast condition selected for the analysis was high TIL against low TIL with the objective of obtaining the genes that were differently expressed between these conditions using a log-likelihood ratio test (LRT) test. A cut-off of 0.05 (adjusted p-value) and 0.1 (absolute log2-fold-change) was used to filter the results. The differential expression analysis was performed globally (all patients) and individually (each patient).

Enrichment analysis was performed with pathfindR (26) using GO-BP (Gene Ontology-Biological Process) and KEGG (Kyoto Encyclopedia of Genes and Genomes) ontologies. Only the differently expressed genes that passed the aforementioned cut-offs were used.

## Results

**Figure 1:**
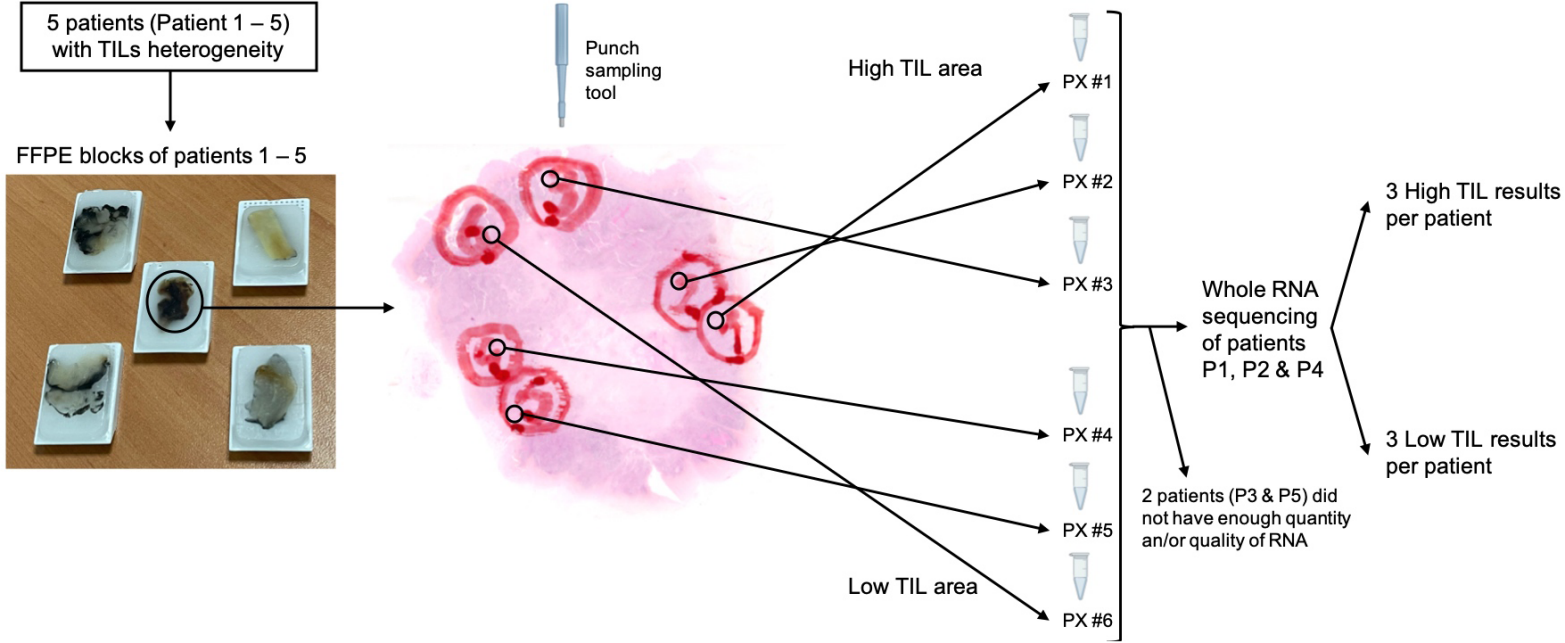
Study schematics (images of the tumour area from Patient 4). PX corresponds to patient’s number and # the triplicates (#1-3 from the high TIL area, #4-6 from the low TIL area). Note: this figure is a representation of the method used, not an example of the exact tissue extracted in one of the patients. TIL, tumor infiltrating lymphocytes; FFPE, formalin-fixed paraffin-embedded.

Five patients were identified to have heterogeneous immune infiltration (a sixth one was also selected but ruled out because its low TIL area was too small to perform triplicates). We performed punches per area in triplicates using a 1.5mm punch sampling tool through the entire thickness of the block (approximately 0.5cm) and isolated the RNA (**Figure 1**). After the punches were performed, we cut ten slides of 4μm to confirm that the heterogeneity was maintained in the following layers of the paraffin block. Only three patients (patient 1, 2 and 4) had enough RNA quality and quantity to perform bulk RNA sequencing in all the triplicates. The clinical and anatomopathological information of the five patients are shown in **Table 1**. Interestingly, patient 1 and 3 had differences in the intratumour histology that matched with the TIL content: the area with higher number of TILs had larger tumour nests, in comparison to the area of low TILs, whose tumour nests were small. Patient 5 had a very large stroma, in many areas it was much wider than the tumour nests.

**Table 1:** Patient’s clinical and anatomopathological characteristics.

# Patient	TNM	Size (mm)	Histology and grade	Low TIL area score	High TIL area score	Overall TIL score
Patient 1	pT2N0M0	32	IDC G2	5%	80%	50%
Patient 2	pT2N0M0	26	IDC G3	5%	50%	30%
Patient 3	pT2N0M0	35	IDC G3	0%	65%	40%
Patient 4	pT1cN0M0	18	IDC G3	<5%	50%	25%
Patient 5	pT2N0M0	35	IDC G3	0%	50%	20%

TIL, tumor infiltrating lymphocytes; TNM, tumour-node-metastasis; IDC, invasive-ductal carcinoma; G, grade.

### Intratumor TIL heterogeneity exhibits different transcriptional profiles

**Figure 2:**
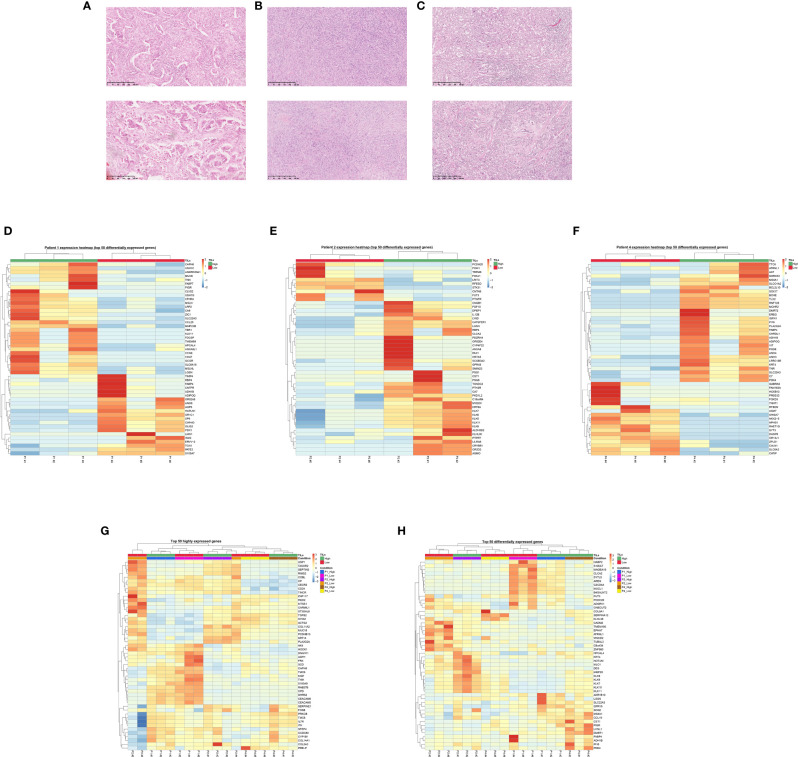
RNAseq reflects intratumor heterogeneity in areas with different TIL content. P1, P2 and P4 represents patient’s number, high TIL areas are marked with #1-3 and low TIL areas with #4-6. **(A–C)** Example of the high (above) and low (below) TIL area of patient 1 **(A)**, 2 **(B)** and 4 **(C)**, 10x. **(D–F)** Heatmap of patient 1 **(D)**, patient 2 **(E)** and patient 4 **(F)** of the 50 genes more differentially expressed per normalized fold-change and adjusted p-value. The triplicates per TIL region cluster together and show a distinct profile versus the other region. **(G)** Heatmap of the 50 highly expressed genes in all the samples, in which each patient´s TIL high or low regions also cluster together in all patients except patient 2. **(H)** Heatmap of the 50 more differentially expressed genes per normalized fold-changed and adjusted p-value. The triplicates from each area from the same patient cluster together.

The location of the punches in the tumor samples of these three patients is shown in **Figures 2A–C**. In **Figures 2D–F**, the top 50 more differentially expressed genes of each patient are shown, in which we can observe that the triplicates of each area cluster together even though sometimes are located far from each other in the tumor sample. There are genes differentially expressed related to immune cells, immune checkpoint receptors, cytokines and their related pathways, but there are also other genes related to the enzymes and proteins present in the extracellular matrix and stroma stiffness like metalloproteinases *(MMP23B)*, metalloproteinase inhibitors *(TIM4)*, keratins *(KRT4)*, kallikreins *(KLK5, KLK6, KLK7, KLK8 and KLK11)*, collagens *(COL14A1, COL5A3* or growth factors (*TGFβ2, FGF10)*. There are also differences in the expression of epithelial markers *(MUCL1, S100A7, CEACAM5, CEACAM6)*, gene regulators *(TBR1*, *SP9*, *TASOR2)* and oncogenes *(MUC16*, *MUC6, BCL2L10)*.

**Figures 2G, H** shows the heatmap of the top 50 expressed genes, and the top 50 differentially expressed genes, respectively. In these two heatmaps, the triplicates of the low or high TIL area from a patient cluster together except for patient 2 for the top 50 expressed genes, in which a triplicate of the low TIL area (P2 #4) clustered with the high TIL triplicates. An explanation for this could be the P2 #4 punch was performed very close to the border, where there were some peripheral lymphocytes that could have been sampled. In **Figure 2G**, the similarity between samples from the same patient prevails over intratumour heterogeneity because the differences between patients in terms of gene expression is higher over the differences between areas. In **Figure 2H** we are representing the differential gene expression and this is why we see that the samples segregate better due to TIL content.

Important oncogenes like *MUC16*, *MUCL1* or *TGFβ2* are more expressed in the low TIL areas of the different patients. MUC16 (mucin 16, also known as CA-125) is a known biomarker for ovarian cancer, which has been shown to play a role in enabling tumor growth and metastasis in many tumors. We observed *MUC16* to be highly expressed in patients 2 and 4 with differences between the low and high TIL area (**Figure 2G**). MUCL1 (mucin like 1, also known as SBEM, Small Breast Epithelial Mucin) seems to have a role in promoting epithelial-to-mesenchymal transition (27), and we observed that is more expressed in the low TIL area of patient 1 (**Figure 2F**). TGFβ2 (transforming growth factor beta 2) is an abundant protein of the extracellular matrix that binds to TGFβ receptors for the recruitment and activation of SMAD family transcription factors that regulate gene expression. The overexpression of TGFβ is known to increase tissue fibrosis because it induces the production of protease inhibitors that prevent the breakdown of the extracellular matrix. The disruption of the TGFβ2/SMAD pathway is implicated in various cancers. *TGFβ2* seems to be more expressed in the low TIL area of patients 2 and 4 (**Figure 2G**).

### Areas with different TIL content exhibit different biological pathways

**Figure 3:**
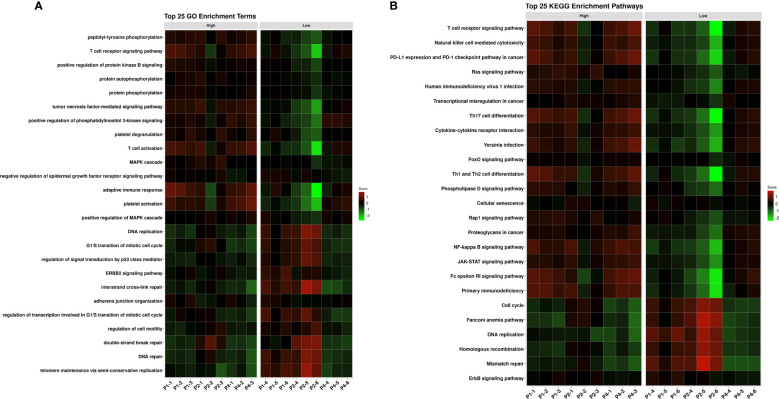
Pathway analyses of differentially expressed genes between high (1-3) and low TIL areas (4-6) of the three patients (P1, P2 and P4) shows activation of different oncogenic, non-immune related pathways. **(A)** Top 25 GO enrichment terms; **(B)** Top 25 KEGG enrichment pathways. TILs, tumor infiltrating lymphocytes; GO, Gene ontology; KEGG, Kyoto encyclopedia of genes and genomes.

GO-BP and KEGG pathway enrichment analyses were conducted to interrogate differences in biological pathways between the differentially expressed genes of high and low TIL area. The top 25 GO and KEGG pathways from differentially expressed genes were shown in **Figures 3A, B**. As expected, pathways related to the activation of immune cells and immune checkpoint inhibitors were upregulated in the high TIL area of the patients. Moreover, MAPK, ERK1 and ERK2 cascade, phospholipase D, calcium and Rap1 signaling pathways were also upregulated in the high TIL area of the patients. By contrast, the low TIL areas had upregulated the ERBB2 signaling pathway, DNA replication, double strand, mismatch and DNA repair.

## Discussion

How the tumor interacts with its microenvironment and the immune system is key to understand the mechanisms employed by the cancer cells to evade immune recognition and elimination. Although this is a small study with a small sample size, to our knowledge, this is the first study that explore differences in the immune infiltration within the same patient, avoiding patient interheterogenity. We have shown that the presence or absence of TILs in patients might be a consequence of the tumor heterogeneity. This difference could be explained due to the tumor intrisic characteristics (for example, their HLA phenotype having different affinity for antigens). However, here we have shown that it may depend also on the tumor heterogeneity. Secretion of different proteins and enzymes, transcriptional alterations, and expression of different regulatory genes and oncogenes by the tumor cells can led to immune recognition and infiltration in some areas but not in others. This observation is reflected in the differential expression analysis in each patient as well as in the gene set enrichment analysis, in which, on top of the upregulation of the immune pathways in the high TIL areas, there is an upregulation of other non-immune related pathways. We hypothesized that the differences in the gene expression between the two areas are due to intrinsic differences between the tumor cells of each area that evolve differently during tumor development.

In the high TIL area, we observed upregulation of pathways related to the immune system such as T cells, Natural Killer cells, PD-1/PD-L1 axis, JAK-STAT, NF-kappa B signaling, cytokines and adaptive immune response, but also upregulation of non-related immune system pathways such as Pi3K, Ras, FoxO, phospholipase D and Rap1 signaling. Pi3K pathway activation has been shown to cause immune evasion through promoting the recruitment of myeloid cells and decreasing CD8+ T cells (28). In the case of low TIL areas, regulation of the signal transduction by p53 class mediator, cell cycle, DNA repair, mismatch repair, homologous recombination and ERBB2 signaling pathways were increased in comparison to the high TIL area. This may indicate that the tumor cells in the low TIL areas are generating more mutations and the cells are trying to repair the DNA, maybe because they are growing faster. It could also be that they are trying to promote pro-apoptotic signals through p53 pathway due to this fast growth and aberrant mutations. Other studies suggest that more adequate DNA repair in some tumor areas is associated with low TIL and that impaired DNA repair would lead to more recruitment of immune cells to the tumor (29). These opposing results could be due to differences in tumour type and disease stage. The current results are consistent with our previous work, where we compared recently diagnosed TNBC patients with high vs. low TILs, and observed numerically more mutations and neoantigens in the low TIL group, being the neoantigen difference only the one statistically significant (21). In this study, we concluded that the lymphocytes in patients with high TILs are eliminating tumour cells in a process called immunoediting, where the most immunogenic clones are being eradicated and only those who can hide from the immune system survive. For this reason, we observed less mutations and neoantigens in these patients than in the low TIL group, where there is no immunological pressure in the tumour microenvironment to control tumour growth.

This study has some limitations that could be addressed in future research. We could only identify five potential cases, and only in three of them we were able to perform RNAseq due to the poor RNA quantity and quality in the other samples. This is partially explained by the fact that it is rare to find patients with such as significant heterogeneous immune infiltration, something that requires a throughout screening process in order to identify more cases, and also because in the last decades the use of neoadjuvant treatment has become the standard of care in TNBC, even for small tumours. It is very likely that if many patients undergo surgery without neoadjuvant treatment, we would have more possibilities to find a higher number of patients with heterogeneous infiltration. There was also a technical limitation with the punches; we were only able to confirm that the heterogeneity was maintained in the following layers but not through all the thickness of the punch. Additionally, it would have been of interest to have performed whole DNA sequencing to study differences in mutations between the two areas, but the tissue left to do this was scarce. Finally, it would have been convenient to perform single cell RNA sequencing, because it can provide a head-to-head comparison between the tumor cells, fibroblasts and stromal cells of the different areas with immune infiltration. It would also allow to observe the expression of the different immune cells in the high TIL area. However, currently, this technique is only compatible with fresh frozen tissue, but the use of FFPE samples may be available in the future. In order to have fresh tissue available for this purpose, it would have been necessary to collect high numbers of fresh frozen samples prospectively because patients with heterogeneous infiltration are very rare, and a H&E staining for TIL assessment to finally identify these patients.

It would of interest for future studies to include TNBC patients in a more advanced disease stage (N1-N3 M0) and see if they are comparable to our patients with no lymph nodes affected. Also, it may be relevant to include another cohort of HER2+ patients with both N0 and N1-N3, since there is a similar proportion of patients with high immune infiltration as the TNBC tumours. Only a very small number of ER+ patients are able to recruit lymphocytes within the tumour, so it is less likely to find patients with heterogeneous immune infiltration in this subtype. Ideally this studies should be done in pre-treatment samples, so that we can compare the natural recognition of the immune system in the onset of the disease without human intervention, which may be key in predicting recurrences after locoregional therapy. Furthermore, this approach can be also expanded to other tumour types such as melanoma or lung, where the immune infiltration is very relevant and being immunotherapy an important treatment option. Since the TILs working group has also published guidelines on how to score TILs in other tumour types different than breast (30), a study with the same methodology as this one can be done in other tumour types.

In conclusion, patients with heterogeneous intratumor immune infiltration show distinctly gene expression profiles, not only upregulation of immune related pathways in the high TIL zone but also the expression of other signaling pathways in both areas that indicates a tumor heterogeneity driving tumor evolution and therefore immune infiltration. This observation warrants further investigation with single cell sequencing between areas with low and high immune infiltration in the same patient to compare the transcriptomics of tumor, fibroblasts and stromal cells between each area. Considering that, in this approach, HLA phenotype, germline DNA and immune repertoire differences are excluded as tumors from different areas come from the same patient. Deciphering the mechanism of the different immune infiltration during tumour growth will give insight into the biology of the intratumor evolution, mechanisms of response and resistance to the immune attack, and identification of biomarkers of response. This could potentially lead to the discovery of new therapeutic strategies to induce immune attraction in patients with low TILs, and mechanisms to reactivate the immune system in patients with high TILs.

## Data availability statement

The datasets presented in this study can be found in online repositories. The names of the repository/repositories and accession number(s) can be found below: https://ega-archive.org/viaaccession ID: EGAS00001007159.

## Ethics statement

The studies involving human participants were reviewed and approved by the Ethics Committee of Vall d’Hebron Hospital in Barcelona, Spain. The patients/participants provided their written informed consent to participate in this study.

## Author contributions

JC and JA conceived the experimental design, designed the inclusion and exclusion criteria for the study and decided the methodology. AQ, EA and CB provided input into the experimental design. AQ, VP and CB identified research subjects, obtained samples and valuated TILs. TM assisted with preparation and processing, JN and JG analyzed and data and designed figures. AQ, EA, CB, JA and JC interpreted data. AQ, EA, JN and JC wrote the manuscript. PS, GC, MM and JA assisted with manuscript writing. All authors contributed to the article and approved the submitted version.
